# Dynamic loading of electrospun yarns guides mesenchymal stem cells towards a tendon lineage

**DOI:** 10.1016/j.jmbbm.2014.07.009

**Published:** 2014-11

**Authors:** L.A. Bosworth, S.R. Rathbone, R.S. Bradley, S.H. Cartmell

**Affiliations:** School of Materials, The University of Manchester, Oxford Road, Manchester, M13 9PL, UK

**Keywords:** Electrospinning, Tendon, Poly(ε-caprolactone), Dynamic loading, Cyclical loading, Mesenchymal stem cells

## Abstract

Alternative strategies are required when autograft tissue is not sufficient or available to reconstruct damaged tendons. Electrospun fibre yarns could provide such an alternative. This study investigates the seeding of human mesenchymal stem cells (hMSC) on electrospun yarns and their response when subjected to dynamic tensile loading. Cell seeded yarns sustained 3600 cycles per day for 21 days. Loaded yarns demonstrated a thickened cell layer around the scaffold׳s exterior compared to statically cultured yarns, which would suggest an increased rate of cell proliferation and/or matrix deposition, whilst maintaining a predominant uniaxial cell orientation. Tensile properties of cell-seeded yarns increased with time compared to acellular yarns. Loaded scaffolds demonstrated an up-regulation in several key tendon genes, including collagen Type I. This study demonstrates the support of hMSCs on electrospun yarns and their differentiation towards a tendon lineage when mechanically stimulated.

## Graphical abstract

**Figure:**
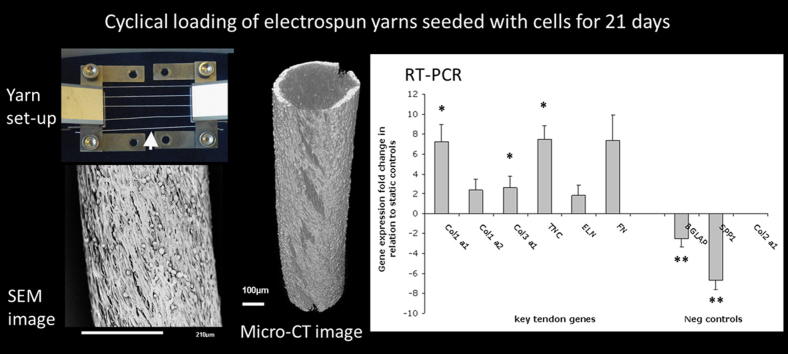

## 1 Introduction

It has long been known that cells respond to mechanical stimuli and that this directly affects their local tissue physiology (Wang and Thampatty, 2006). This is also the case for *in vitro* studies, where mesenchymal stem cells (MSCs) have been driven towards a vascular smooth muscle cell phenotype following their cyclical stretching on flexible, silicone membranes causing an increased expression of smooth muscle contractile markers (Park et al., 2004); and repeated compressive loading of MSCs resulting in raised levels of aggrecan and glycosaminoglycans, indicative of their differentiation towards a chondrocyte/cartilaginous phenotype (Mauck et al., 2007). Similar studies have been performed using tendon fibroblasts and MSCs in order to stimulate production of tendon-like tissue *in vitro* for tissue engineering applications (Garvin et al., 2003, Cao et al., 2006, Nirmalanandhan et al., 2007, Issa et al., 2011, Teh et al., 2013).

Tendons are a type of connective tissue, capable of withstanding high tensile loads to enable movement. They are susceptible to injury caused by wear and tear or spontaneous rupture. A segmental repair or reconstruction of the tissue may be required depending on the type of injury incurred. In cases like this, surgeons will graft autologous tissue taken from a secondary site. However, problems can arise when the patient does not have sufficient and/or adequate tissue to harvest as a graft. This lack of usable tissue has driven researchers in the biomaterials and tissue engineering field to develop alternative replacement graft strategies.

In an innovative study, Garvin et al. (2003) fabricated bioartificial tendons using tendon fibroblasts (sourced directly from avian flexor tendons) suspended in collagen type I gels that were subjected to cyclical loading using a Flexcell Tissue Train system. A loading regime of 1 h per day at 1% strain and frequency 1 Hz, was sufficient to generate changes in gene expression levels. Where a number of key tendon genes, such as collagen Types I, III and XII, fibronectin and tenascin, were expressed at levels similar to those within natural avian flexor tendon tissue. After stimulation for 7 days, the mechanical strength of the bioartificial tendons was almost three times stronger compared to the non-loaded control group, but remained significantly weaker than the native tendon. Cao et al. (2006) conducted a study whereby tendon fibroblasts were seeded onto unwoven polyglycolic acid (PGA) fibres that were mounted on a U-shaped spring for six weeks. In the control group, cell-seeded fibres were cultured strain-free, and in the test group the spring had a constant strain applied. Their results showed it was possible to generate tendon tissue and that the tissue structure matured and strengthened significantly over time when a constant strain was applied. However, continuous strain affected the morphology of the formed tissue as the collagen fibres appeared compacted when compared to natural tendon tissue and the authors surmised that applying a constant tension was not appropriate for this type of tissue engineering, which was aiming to replicate the natural physiology. More recently, Teh et al. (2013) investigated the synergistic effects of mechanically stimulating aligned silk fibroin hybrid scaffolds seeded with MSCs. A loading pattern of 12 h per day, frequency 0.1 Hz and 5% translational strain and 90° rotational strain were applied for 11 days. Their findings determined tenogenesis to be enhanced for scaffolds held under dynamic culture conditions as gene expression levels, including collagen Type I, tenascin-C and tendomodulin, were up-regulated compared to static controls. In terms of mechanical properties, the loaded scaffolds were similarly found to possess greater tensile strength than their static cultured counterparts.

**Fig. 1:**
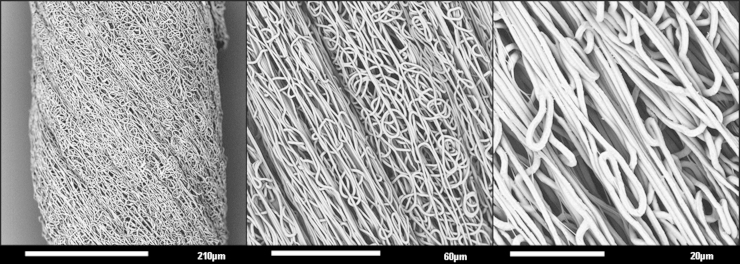
Scanning electron micrographs demonstrating the structure and surface topography of the electrospun yarn without cells at increasing magnifications.

A common opinion in biomaterials and tissue engineering is to produce scaffolds that mimic the architecture of the natural tissue as closely as possible (Ma et al., 2005, Vasita and Katti, 2006). As such, electrospinning has become a widely used technique to easily produce fibrous scaffolds with structures reminiscent of a tissue׳s extracellular matrix (Wang et al., 2013). Capable of supporting a wide range of cell types, electrospun scaffolds have been investigated for the repair and regeneration of bone (Yang et al., 2013), nerves (Koh et al., 2010), bladder (Stankus et al., 2008), amongst many others. Electrospun scaffolds that possess a parallel arrangement of fibres are currently being researched for the repair of damaged tendons (Bosworth et al., 2013). In this case, three-dimensional electrospun fibrous yarns – a continuous strand of twisted fibres – were found to be superior scaffolds compared to the more common two-dimensional sheets of aligned fibres for this particular tissue type. This study builds on this previous research and investigates the effects of cyclically loading electrospun yarn (Fig. 1) cultured with and without hMSCs, in order to determine if the yarns provide a suitable topography and structure to support hMSCs and transfer the mechanical stimulus to the cells to instigate a change in phenotype.

## 2 Materials and methods

### 2.1 Electrospinning of scaffolds

A 10%w/v solution of poly(ε-caprolactone) (PCL; Purac Purasorb PC12 (*M*_*w*_ 120,000 g/mol) dissolved in 1,1,1,3,3,3-hexafluoroisopropanol (HFIP; Sigma) was electrospun using parameters: voltage – 20 kV, flow-rate – 1 ml/h, distance to collector – 200 mm, spinning time – 15 min. Fibres were collected on the edge of a mandrel (Ø 120 mm, width 3 mm) rotating at 600 RPM, and removed as a single fibrous ribbon.

Electrospun yarns were fabricated by cutting the fibrous ribbon into 50 mm lengths and then briefly submerging in distilled water. The strips were then manually twisted along their lengths to create yarns of electrospun fibres (Ø ~200 µm) (as previously described in Bosworth et al., 2013).

### 2.2 Sterilisation of electrospun yarns

Yarns were sterilised in increasing concentrations of ethanol (VWR) in distilled water (50, 70, 90, 100%v/v; 24 h per concentration), and then washed twice in Phosphate Buffered Saline solution (PBS) (Invitrogen; 12 h per wash). Following washing with PBS, yarns were submerged in mesenchymal stem cell culture medium with supplement mix (PromoCell) and 1% antibiotic (penicillin/streptomycin; Gibco).

### 2.3 Experimental set-up

**Fig. 2:**
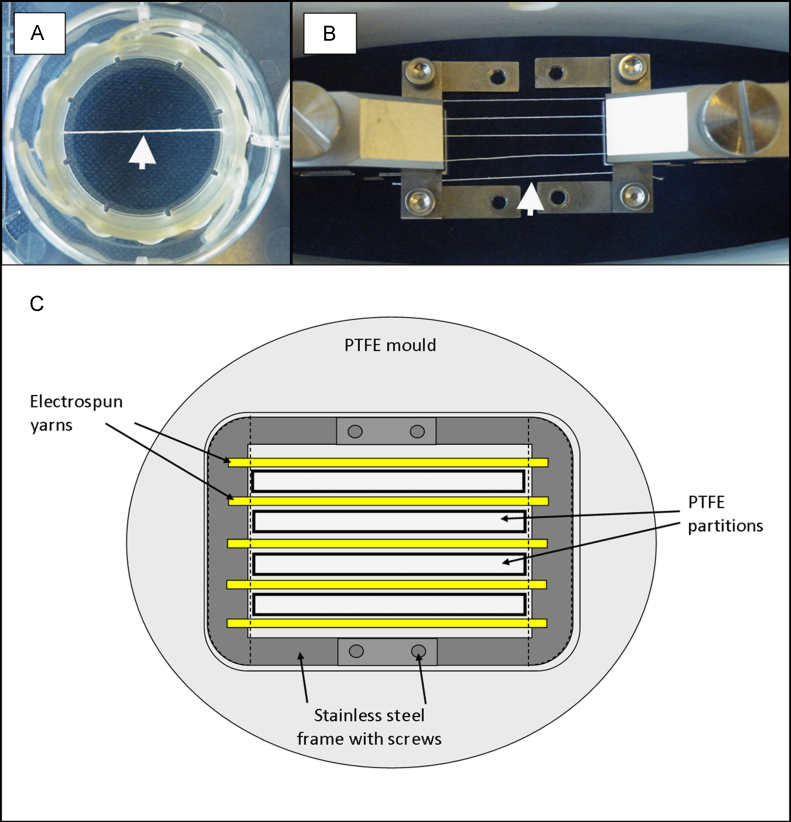
Experimental set-up: (A) single electrospun yarns securely anchored within 6-well CellCrowns and placed within low binding well plates (arrow highlights position of yarn). (B) Five electrospun yarns held within a custom-made stainless steel frame and loaded between the grips of the BOSE biodynamic chamber (arrow highlights position of yarn). (C) Diagram demonstrating the position and separation of electrospun yarns within the frame and PTFE mould with partitions.

Yarns were subjected to either static or cyclical loading conditions and seeded either with cells or kept acellular. Those to be kept static were mounted into 6-well CellCrowns (Scaffdex) and placed within low binding, 6-well culture plates (Corning) (Fig. 2a). Yarns subjected to cyclical loading were mounted into a custom-made frame, which could hold 5 yarns at one time (Fig. 2b).

### 2.4 Cell seeding

For cell seeding, the bespoke frame was initially placed within a custom-made PTFE mould with partitions that allowed each yarn to be seeded separately (Fig. 2c). Primary human mesenchymal stem cells derived from bone marrow (60 year old, Caucasian female; PromoCell) were cultured to passage 4 and seeded at a concentration of 500,000 cells in 200 µl per scaffold. Cells were left to adhere and acclimatise to the scaffolds for 5 days in MSC culture media at 37 °C, 5% CO_2_.

### 2.5 Experimental start

Following 5 days, the frame was removed from the mould and mounted within the BOSE biodynamic chamber (BD5110) with 225 N load cell and subjected to a tensile, sinusoidal loading regime (5% strain, 1 Hz for 1 h per day; 3600 cycles per day). Yarns held within CellCrowns and low binding well plates remained under static conditions with zero strain. All electrospun yarns were fully submerged in MSC culture media at 37 °C, 5% CO_2_ for 7 and 21 days. Cell media was changed every three days.

### 2.6 Yarn and cell morphology

After 7 and 21 days in culture, yarns were washed in PBS and fixed in 1.5%v/v glutaraldehyde (TAAB Laboratories) in PBS for 30 min at 4 °C. Samples were rinsed twice in PBS and then dehydrated through increasing concentrations of ethanol in distilled water (50–100%v/v), followed by chemical drying in hexamethyldisilazane (HMDS; Sigma). The samples were gold sputter-coated for 2 min and visualised by Scanning Electron Microscopy (SEM) (Phenom Pro) operating at 5 keV.

### 2.7 Cell infiltration

Cell infiltration was investigated in 3D by using high resolution X-ray tomography. After 21 days, yarns were washed in PBS and fixed in 1.5%v/v glutaraldehyde (TAAB Laboratories) in PBS for 30 min at 4 °C. Samples were washed twice in PBS and then placed in osmium tetroxide (Agar Scientific) (1% in water) and incubated for 15 min in the dark at room temperature to provide an increased X-ray contrast of the cells compared to the inherent contrast of the scaffold. Yarns were rinsed five times with PBS and then dehydrated through increasing concentrations of ethanol in distilled water (50–100%v/v), followed by chemical drying in HMDS. Yarns were mounted vertically within polyimide tubing (Cole-Parmer) with 1 mm diameter, which was then sealed at either end. Yarns were scanned vertically within a Xradia microXCT-400 with source voltage 40 kV, power 10 W, and 20× objective. 721 radiographs were taken over 182 degrees, with scan time of ~12 h. 3D volumetric data was reconstructed using the FDK (Feldkamp–Davis–Kress) algorithm, resulting in final voxel size of 0.61 μm. Scans were visualised using VG Studio Max v.2.0.

### 2.8 Gene expression

At each time point RNA extraction and reverse transcription was carried out using a MicroMacs one-step cDNA kit (Miltenyi Biotech Ltd) to generate cDNA. Real-time (RT)–PCR was performed with a step-one PCR system, model v2.1 (Applied Biosystems). TaqMan Gene Expression Assays (Applied Biosystems) were performed specific for key genes expressed in the formation of tendon tissue, along with negative controls for osteogenesis and chondrogenesis, and GAPDH as the house keeping gene (*n*=4) (Table 1). Relative gene expression levels were calculated using the delta–delta Ct method. The data was expressed as the mean±standard deviation; *n*=4 with unpaired two-tailed student *T*-test.

**Table 1:** List of genes investigated, coupled with their associated code for Taqman probes.

**Gene of interest**	**Taqman probe code**
**Tendon tissue**	
Collagen Type I (Col1a1 and Col1a2)	Hs00164004_m1 Hs00164099_m1
Collagen Type III (Col3a1)	Hs00943809_m1
Tenascin-C (TNC)	Hs01115665_m1
Elastin (ELN)	Hs00355783_m1
Fibronectin (FN)	Hs00355783_m1

**Negative controls**	
Osteopontin (SPP1)	Hs00167093_m1
Osteocalcin (BGLAP)	Hs00609452_g1
Collagen Type II (Col2a1)	Hs01064869_m1

**House keeping gene**	
GAPDH	Hs99999905_A

### 2.9 Tensile testing

At each time point, yarns were immediately tensile tested to failure using an Instron 1122 with 10 N load cell and 5 mm/min crosshead speed. Yarns were kept under wet conditions prior to performing the tensile test. Number of samples per group=5, a Mann–Whitney test was used for statistical analysis with 95% confidence level.

### 2.10 Yarn crystallinity

Acellular yarns were washed in PBS and vacuum dried at room temperature for 24 h. Yarns were individually sealed in aluminium pans and placed within the Differential Scanning Calorimeter (DSC) where they were exposed to a single heat cycle from 0 to 100 °C with 10 °C/min heating rate (DSC Q100; TA Instruments, version 9.9). Nitrogen gas flow was 50 ml/min. Universal Analysis 2000.v.4.2E software (TA Instruments) was used to quantify the Enthalpy of Fusion (∆*H*_m_). Material crystallinity was determined by comparing ∆*H*_m_ with that for 100% crystalline PCL (135.44 J/g), as measured by Crescenzi et al. (1972). Yarns were tested in triplicate and statistically analysed using one-way ANOVA with Bonferroni post-tests and 95% confidence level.

## 3 Results

### 3.1 Cell morphology

**Fig. 3:**
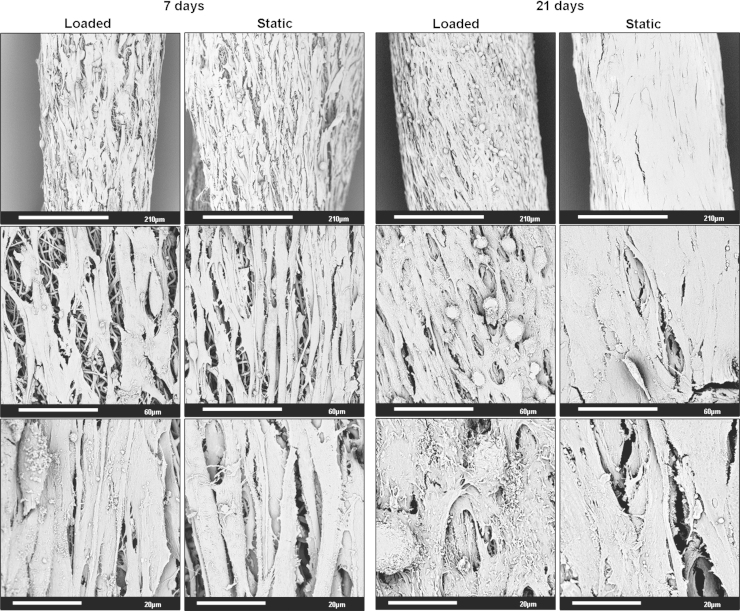
Scanning electron micrographs of electrospun yarns seeded with human mesenchymal stem cells that have been cultured for 7 and 21 days, and subjected to either cyclical loading or kept under static conditions.

Irrespective of loading regime, the hMSCs adhered and proliferated over the surface of the electrospun yarns (Fig. 3). Similarly, the orientation of the cells appeared to be governed by the underlying fibre direction as cells were arranged parallel to these fibres regardless of being subjected to static or cyclical conditions. After 21 days, the surfaces of the yarns were confluent with cells; however, hMSCs on the cyclically loaded yarns were more textured and round compared to those on the statically cultured yarns, which appeared to be flatter and fused together.

### 3.2 Cell infiltration

**Fig. 4:**
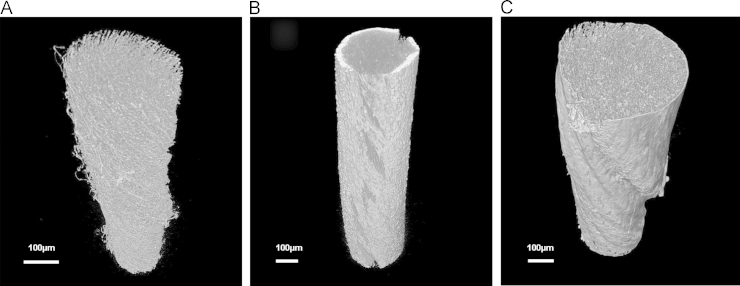
Micro-CT scans of (a) acellular electrospun yarn at time zero; (b) electrospun yarn with human mesenchymal stem cells (hMSCs) subjected to dynamic loading for 21 days; (c) electrospun yarn with hMSCs held for 21 days under static conditions. Scale bar=100 µm.

Migration of hMSCs into the yarn׳s core was assessed by microXCT (Fig. 4). An initial scan of the acellular yarn was performed and total porosity was determined to be 56%. Scanning the yarns with cells after 21 days revealed clear differences. Cells subjected to cyclical loading possessed a thick layer of cells, approximately 30 µm deep, around the outer circumference of the yarn. In comparison, cells cultured under static conditions demonstrated no thickened layer. Both test groups highlighted adhesion of cells around the entire outer yarn surface, but there was no clear evidence of cell infiltration into the structure.

### 3.3 Gene expression

**Fig. 5:**
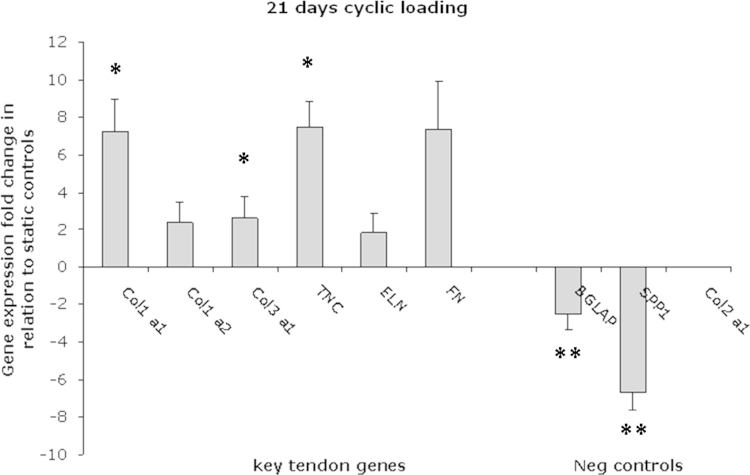
Gene expression analysis of human mesenchymal stem cells demonstrating fold change increase in relation to statically cultured yarns after 21 days. Where Col1a1 and Col1a2 represent Collagen Type I, Col3a1—Collagen Type III, TNC—Tenascin-C, ELN—Elastin, FN—Fibronectin, bone markers—BGLAP and SPP1, and cartilage marker—Col2a1. Unpaired two-tailed student *T*-test (^*^p<0.05,^**^p<0.1).

After 21 days, gene expression levels of seeded cells were quantified (Fig. 5). For cyclically loaded yarns, there was an up-regulation in a number of key tendon genes, collagen I (Col1a1, Col1a2) and III (Col3a1), tenascin-C (TNC), elastin (ELN) and fibronectin (FN), with significance for Col1a1, Col3a1 and TNC. Negative controls were also down-regulated for cyclically loaded yarns, which proved these MSCs were not differentiating down a bone/cartilage lineage.

### 3.4 Tensile properties

**Fig. 6:**
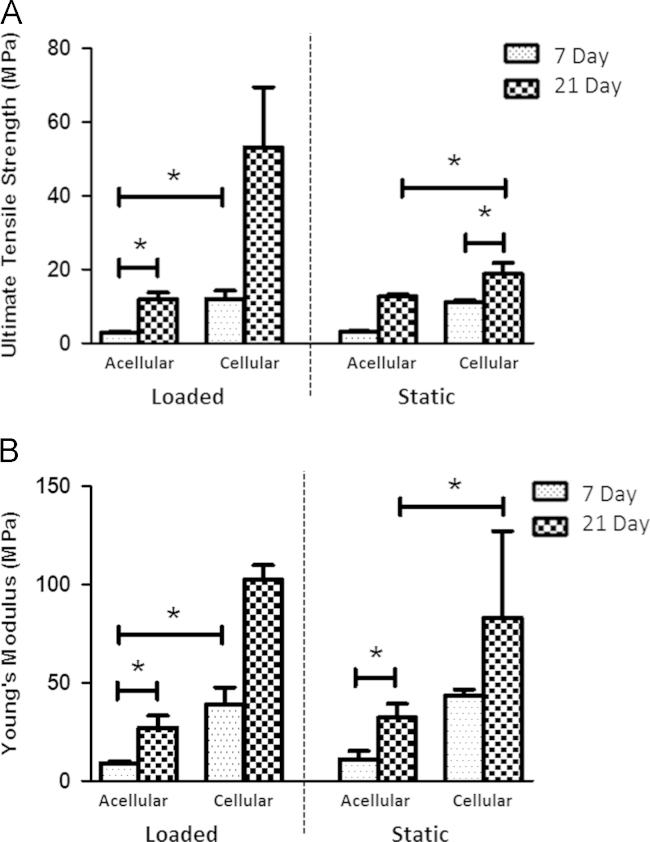
Tensile testing data for acellular and cell-seeded yarns subjected to either cyclical loading or held under static conditions. Where (a) demonstrates Young׳s Modulus and (b) ultimate tensile strength. Mann–Whitney test (^*^p<0.05).

Yarns were tensile tested to failure for acellular and cellular, static or loaded groups (Fig. 6). A number of significant observations were apparent. Irrespective of loading regime and presence of cells, tensile strength and stiffness of the yarns increased with time. Strength and stiffness similarly increased when cells were present compared to their acellular counterparts. There were no significant differences in tensile properties between loaded *versus* static cohorts at either time-point.

### 3.5 Thermal properties

In the time frame investigated the crystallinity of the yarns did not change significantly and ranged between 25 and 33% (Table 2). Similarly, there was no notable difference in crystallinity depending on which loading regime was applied.

**Table 2:** Enthalpy of Fusion and percentage crystallinity data for acellular yarns subjected to loading or static conditions for 7 and 21 days. Data shown as mean±standard deviation; statistical analysis using one-way ANOVA with Bonferroni post tests.

Sample	Enthalpy of Fusion (J/g)	Crystallinity (%)	*p* value
Static 7 day	44.92±0.73	33.16±0.54	Not significant
Loaded 7 day	38.27±4.13	28.26±3.05	
Static 21 day	38.05±0.19	28.09±0.14	
Loaded 21 day	34.51±9.16	25.48±6.76	

## 4 Discussion

Electrospun fibre scaffolds commonly support the attachment and proliferation of many cell types and this remains the case for electrospun fibre yarns seeded with hMSCs. Electrospun fibres have long been known to confer contact guidance cues to adhering cells (Bosworth et al., 2012). As such, the orientation and elongation of these hMSCs is controlled by the direction of the underlying fibres and is not affected by mechanical stimulus, which is in agreement with a recent study by Subramony et al. (2013) who determined MSC differentiation into ligament fibroblast-like cells is driven initially by the underlying surface topography, followed by mechanical stimulation. Application of one cycle per second for 1 h each day did, however, affect cell morphology. After 21 days, cells subjected to loading possessed a different phenotype and appeared to be three-dimensional in structure, shorter in their lengths and presence of several entirely rounded cells visible from the micrographs (Fig. 3). In contrast, their static counterparts displayed a flattened and well-spread shape, with cells appearing merged with no clear separation of their membranes. This would suggest that despite cells actively proliferating on both yarns, cells on the loaded scaffold were sensing the applied mechanical force and it was affecting their morphology. Further experimental work would need to be performed in order to fully understand this observed change and would require a specifically designed bioreactor system that would allow live cell fluorescent imaging, determination of cell motility and assessment of the cell membrane. Optical Coherence Tomography (OCT) could also be used to non-invasively image cells within the yarns (Yang et al., 2006). In the native tissue, tendon fibroblasts lie in columnar rows and fully surrounded by collagen fibres giving a true 3D culture environment. This *in vitro* study, however, does not provide the same 3D surrounding as the cells are positioned on top of the fibrous scaffold, thus it is difficult to draw any conclusions with regard to which loading regime exerts an appropriate cell morphology after this time period.

Scanning electron micrographs demonstrated the exterior surface of both static and loaded yarns to be surrounded by cells after 21 days; however, this analytical technique fails to determine whether cells had infiltrated the scaffold. To determine if cells had been able to migrate through to the yarn׳s internal structure, microXCT was performed (Fig. 4). Initial assessment of the acellular yarn, determined a total porosity of 56%, which indicates the scaffold to be relatively porous and thus should support cell infiltration. It is worth noting, this percentage is likely to be the absolute minimal level of porosity for these scaffolds as the microXCT used in this study was limited to measure pore spaces that were greater than 0.7 µm in width, hence any smaller pores will not have been included. Aligning electrospun fibres is known to reduce pore size compared to random fibre networks because the fibres are in closer proximity to each other (Bosworth and Downes, 2009); similarly the method used to produce these electrospun yarns is likely to further reduce the pore size as the twisting of aligned fibres draws them closer together creating a tight structure. Furthermore the approximate diameter of these hMSCs was 20 µm, which was considerably larger than the void space available. Following scanning of both the loaded and static cell-seeded yarns after 21 days, no infiltration of cells was observed, suggesting all cells remained solely on the scaffolds’ outer periphery. This lack of infiltration could have considerable and detrimental consequences to the long-term success of this yarn should it be implanted as a tissue-engineered device. Without cell and tissue in growth penetrating through to the scaffold core, the device could fail mechanically, either due to a void being left within the tissue following its degradation, or the device could become encapsulated by the surrounding tissue, which may need to be explanted at a later date. Despite this lack of infiltration, however, it should be remembered that this is an *in vitro* study and although every effort is made to best simulate *in vivo* conditions it can still fall short. In a separate study, these electrospun yarns were implanted (without cells) into the flexor digitorum profundus tendons of mice and infiltration of cells to the scaffold core was observed after the same time frame of 21 days (Bosworth, 2013). This could be due to remodelling of the yarn׳s fibres by the surrounding cells and tissue once implanted and/or due to accelerated degradation of the polymer, which could loosen the structure allowing migration of cells into the scaffold structure. Providing this yarn is to be used as an acellular graft and not a tissue-engineered device, there is currently no need to alter its structure at this stage. Although to prove its long-term efficacy, further assessment should be undertaken over an extended time period. If the yarn does require structural alterations to improve its pore size, this could be achieved by incorporating sacrificial fibres made of water-soluble poly(ethylene oxide) (PEO) dual electrospun with the PCL fibres, which when in contact with water would leave a PCL yarn with a more open structure and increased porosity, as demonstrated by Baker et al. (2008). Despite cells not infiltrating the yarns *in vitro*, the microXCT scans further highlighted a clear difference in cell response when subjected to different loading regimes. The outer circumference of scaffolds kept under static conditions were covered by a thin cell layer; whereas yarns subjected to cyclical loading presented a thickened cell layer (~30 µm) around the scaffolds’ exterior. Similar to the change in observed cell morphology, the application of load appeared to affect cell response and may have triggered an increased rate of cell proliferation and/or matrix deposition compared to cells kept under static conditions. A repeated study which assessed the rate of cell proliferation between the two groups would be beneficial to further understand the differences observed at this stage.

Gene expression was quantified after 21 days and demonstrated an up-regulation in a number of tendon-related genes for cell-seeded yarns subjected to cyclical loading, including; collagen (Col1a2), elastin, fibronectin and with significance for collagen (Col1a1 and Col3a1) and tenascin-C (Fig. 5). Healthy tendons are predominantly composed of collagen, with Type I being the major component. Observation of significant levels of collagen for loaded yarns demonstrates the adhering hMSCs are being positively stimulated to secrete matrix of an appropriate composition compared to their static counterparts. Tenascin-C, which is present in developing tendons and following tendon tissue injury, was similarly up-regulated for cyclically loaded cells and further demonstrates the beneficial effect a mechanical stimulus has on guiding these stem cells towards a tendon phenotype. With a number of previous studies demonstrating similar trends with respect to gene expression and loading (Nirmalanandhan et al., 2007, Issa et al., 2011, Teh et al., 2013, Subramony et al., 2013, Barber et al., 2013) this investigation further adds to the importance of applying a mechanical stimulus in order to drive MSC differentiation towards a tendon lineage. In particular, Doroski et al. (2010) reported an identical gene expression pattern at 21 days for human marrow stromal cells – upregulation of collagen types I and III and tenascin-C – cultured within a poly(ethylene) glycol-based hydrogel and subjected to cyclical loading. Despite using different loading conditions (10%, 1 Hz, 3 h of strain followed by 3 h rest), this current study demonstrates a similar response can be obtained using a lower number of cycles per day (3600 as opposed to 86,400 applied by Doroski et al., 2010) and further contributes to the growing body of evidence demonstrating differentiation of cells towards a tendon lineage using different mechanical stimulation.

The tensile properties of the yarns with/without cells and with/without loading were determined at 7 and 21 days (Fig. 6). Irrespective of cells being present and loading over the time period investigated, the tensile properties were found to significantly increase in strength and stiffness. This trend is in agreement with a previous study assessing *in vitro* degradation of a similar scaffold (note different manufacturer, molecular weight and purity of PCL) in PBS (Bosworth and Downes, 2010) and would suggest the yarn has undergone modifications to its internal structure, such as changes in degree of crystallinity. PCL is known to be a semi-crystalline polymer and its amorphous regions are attacked foremost, which can lead to new bonds forming and subsequent increases in the level of crystallisation being observed. However, in this study the crystallinity of acellular yarns remained unchanged when comparing 21 day data with 7 day (Table 2). Possible reasons for the significant changes observed in tensile properties may be attributed to the scaffold holding less water within its structure due to remodelling and shrinkage causing ‘closure’ of the pores with time. This would require further testing to fully elucidate the behavioural changes that may be occurring within the scaffold, such as measurement of creep, scaffold dimensions, wet weight and pore size. A number of papers report decreases in structural strength when tested under wet conditions compared to dry, thus it would be beneficial in future work to similarly compare this effect on the electrospun yarn (Sarasam and Madihally, 2005, Hosaka et al., 2007, Guan and Davies, 2004). Greatest Modulus and strength were obtained for cell-seeded yarns subjected to cyclical loading for 21 days. This trend was observed for all cell-seeded yarns regardless of loading regime, with presence of cells providing a notable improvement in strength and stiffness. This improvement in tensile properties could suggest the deposited cell/matrix layer observed by microXCT has integrated with the outer fibres of the yarn and is providing additional strength to the scaffold; similar to the epitenon, a sheet of connective tissue which encapsulates the collagen fibre fascicles within the tendon and can fuse with superficially located tendon fibrils (Kannus, 2000). Despite demonstrating an increase in strength and stiffness there was no significant difference between yarns that had been loaded *versus* those that had been held under static conditions. This observation is counterintuitive and a trend similar to other published studies, such as, Abousleiman et al. (2008) who reported increases of 156% in strength and 109% stiffness for cell/constructs subjected to cyclical tension compared to static samples, would be expected. A repeat of the study with a larger sample size would be recommended to fully comment on the effects of loading on cell-seeded yarn mechanical properties. The type of material construct may also play a part in the level of mechanostimulation being transferred directly to the cells—a study by Nirmalanandhan et al. (2007) demonstrated a greater improvement in tensile properties for MSCs held within collagen sponges compared to collagen gels. Relating this new data to published figures of tensile tested human tendons, the mechanical properties of these electrospun yarns remains relatively weak; for example, Wren et al. (2001) determined the Modulus and tensile strength of the human Achilles to be 816±218 MPa and 71±17 MPa, respectively. In order to achieve a stiffer and stronger scaffold that can accommodate the size of defect, this electrospun yarn is likely to require braiding of some kind to create larger and stronger woven structures.

## 5 Conclusion

This study investigates the seeding of hMSCs and effects of mechanical stimulation on novel 3D electrospun yarns. Irrespective of loading regime, electrospun yarns supported the adhesion and proliferation of hMSCs and further guided cell orientation to lie parallel to the underlying fibre direction. Dynamic loading of cell-seeded scaffolds appeared to increase the rate of cell proliferation and overall tensile strength of the yarn, and resulted in the up-regulation of a number of key tendon genes, indicating MSC differentiation towards a tendon lineage.
